# Spontaneous regression of transverse colon cancer: a case report

**DOI:** 10.1186/s40792-017-0341-z

**Published:** 2017-05-10

**Authors:** Keigo Chida, Kazuaki Nakanishi, Hiroki Shomura, Shigenori Homma, Atsuo Hattori, Keizo Kazui, Akinobu Taketomi

**Affiliations:** 1 Department of Surgery, Japan Community Healthcare Organization Hokkaido Hospital, Kita-Ku, Kita 14, Nishi 7, Sapporo, 060-8638 Japan; 2 Department of General Surgery, Graduate School of Medicine, Hokkaido University, Sapporo, Japan; 3 Department of Pathology, Japan Community Healthcare Organization Hokkaido Hospital, Sapporo, Japan

**Keywords:** Spontaneous regression, Colon cancer, Immune response

## Abstract

Spontaneous regression (SR) of many malignant tumors has been well documented, with an approximate incidence of one per 60,000–100,000 cancer patients. However, SR of colorectal cancer (CRC) is very rare, accounting for less than 2% of such cases. We report a case of SR of transverse colon cancer in an 80-year-old man undergoing outpatient follow-up after surgical treatment of early gastric cancer. Colonoscopy (CS) revealed a Borrmann type II tumor in the transverse colon measuring 30 × 30 mm. Because the patient underwent anticoagulant therapy, we did not perform a biopsy at that time. A second CS was performed 1 week after the initial examination and revealed tumor shrinkage to a diameter of 20 mm and a shift to the Borrmann type III morphology. Biopsy revealed a poorly differentiated adenocarcinoma. One week after the second CS, we performed a partial resection of the transverse colon and D2 lymph node dissection. Histopathology revealed inflammatory cell infiltration and fibrosis from the submucosal to muscularis propria layers in the absence of cancer cells, leading to pathological staging of pStage 0 (T0N0). The patient had an uneventful recovery, and CS performed at 5 months postoperatively revealed the absence of a tumor in the colon and rectum. The patient continues to be followed up as an outpatient at 12 months postoperatively, and no recurrence has been observed.

**Electronic supplementary material:**

The online version of this article (doi:10.1186/s40792-017-0341-z) contains supplementary material, which is available to authorized users.

## Background

Spontaneous regression (SR) of a malignant tumor is defined as a partial or complete disappearance of a tumor without treatment or in response to a treatment that is considered inadequate to exert a significant influence on neoplastic disease [1]. SR has been well documented for many types of cancer, with an overall estimated incidence of approximately one per every 60,000–100,000 cancer patients [2]. However, SR of colorectal cancer (CRC) is very rare, accounting for less than 2% of such cases [3]. Here, we report a case of SR of transverse colon cancer in which we could morphologically observe the course of shrinkage.

## Case presentation

An 80-year-old man with a previous history of interstitial pneumonia, paroxysmal atrial fibrillation (pAf), and hypertension was undergoing outpatient follow-up after surgical therapy for early gastric cancer. He was not receiving chemotherapy and immunotherapy and was orally taking apixaban only.

**Fig. 1:**
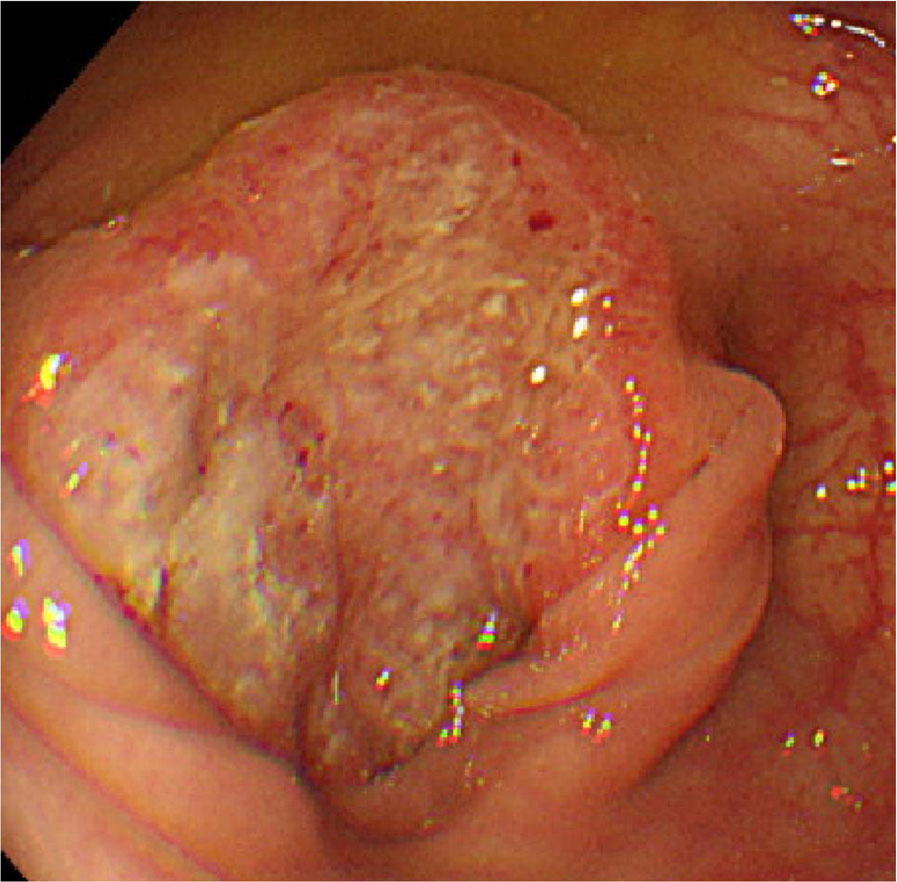
Image from the first colonoscopy (CS). The first colonoscopy (CS) revealed a Borrmann type II tumor with measurements of 30 × 30 mm in the transverse colon

**Fig. 2:**
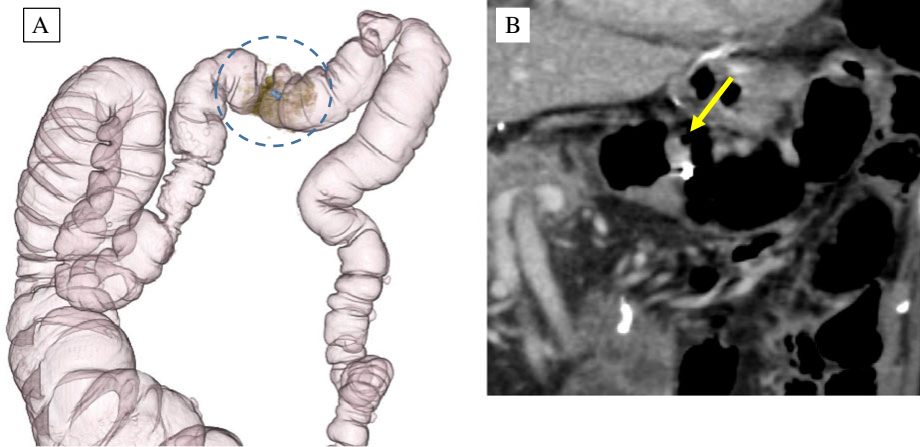
**a**, **b** Images from abdominal enhanced computed tomography (CT). CT revealed an increased wall thickness along the left half of the transverse colon [indicated by a *dotted line* (**a**) and *arrow* (**b**). However, the lack of increased CT values in the surrounding fatty area suggests that the invasion depth was the muscularis propria (MP) layer. No lymph node or distant metastasis was observed

Laboratory evaluations conducted during the third postgastrectomy year revealed an elevated level of the tumor marker carbohydrate antigen (CA) 19-9 (44 U/ml; normal range, <37 U/ml). Although colonoscopy (CS) revealed a Borrmann type II tumor in the transverse colon measuring 30 × 30 mm (Fig. 1), a biopsy was not performed because the patient was undergoing anticoagulant therapy for pAf. Computed tomography (CT) revealed increased wall thickness in the left half of the transverse colon; however, the absence of increased CT values in the surrounding fatty area suggested that its invasion depth was the muscularis propria (MP). No lymph node or distant metastasis was detected (Fig. 2a, b). The clinical staging of the tumor was cStage I (cT2N0M0) according to the TNM classification.

**Fig. 3:**
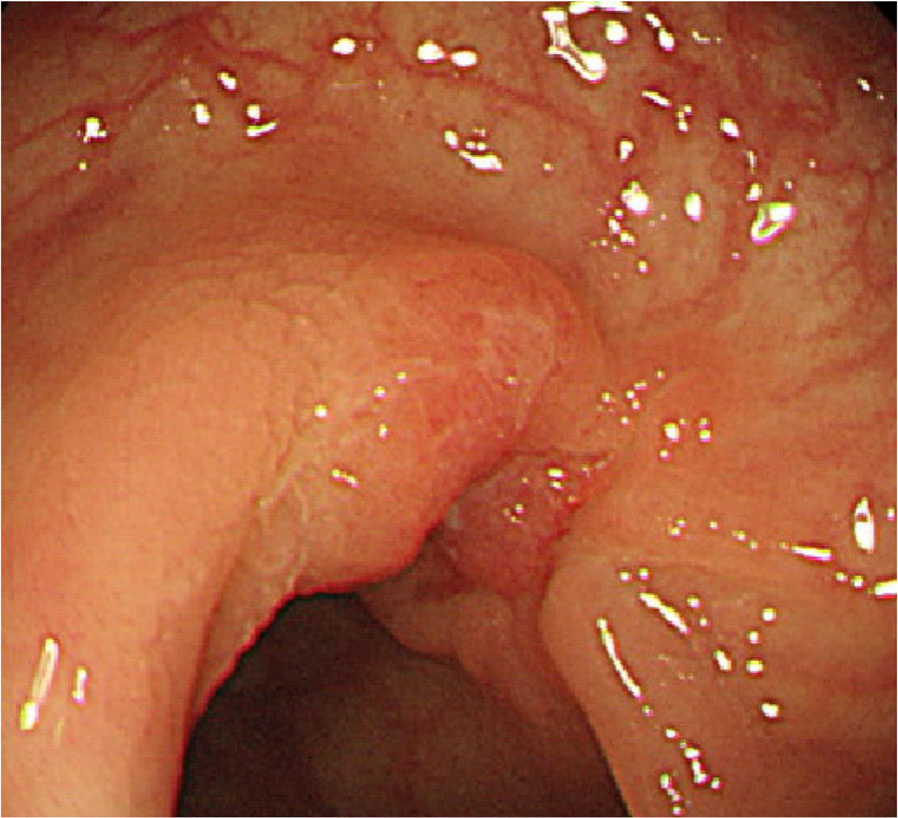
Image from the second colonoscopy (CS). The second CS revealed tumor shrinkage to a diameter of 20 mm and a shift to Borrmann type III morphology

**Fig. 4:**
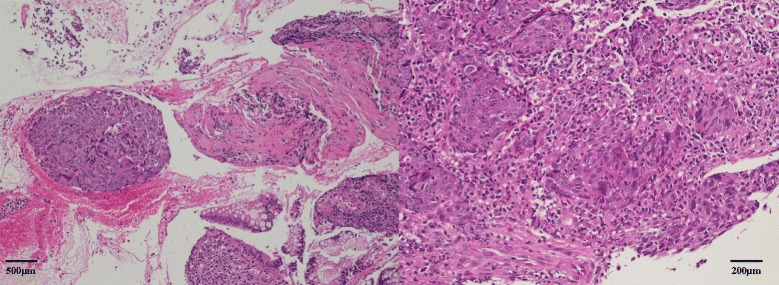
Histopathological examination of the biopsy specimen. Histopathology of the biopsy specimen revealed a poorly differentiated adenocarcinoma surrounded by a significant lymphocytic aggregate

**Fig. 5:**
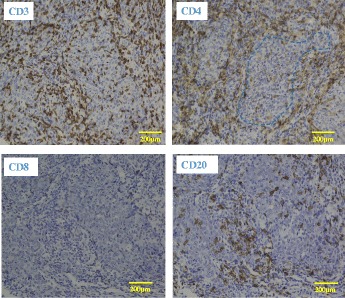
Immunohistochemical staining of the biopsy specimen. Immunostaining revealed that the aggregated cells mainly comprised CD3^+^CD4^+^ T cells and CD20^+^ B cells, with very few CD8^+^ T cells

A second CS was performed 1 week after the initial examination, at which time the tumor exhibited shrinkage to a diameter of 20 mm and a morphological shift to Borrmann type III (Fig. 3). Histopathogical evaluation of the biopsy specimen revealed a poorly differentiated adenocarcinoma surrounded by a significant lymphocytic aggregate (Fig. 4). Immunohistochemical staining of this specimen revealed that the aggregated cells were mainly CD3^+^CD4^+^ T cells and CD20^+^ B cells; CD8^+^ T cells were not observed (Fig. 5).

One week after the second CS, the patient underwent the surgery. Because, intraoperatively, we could not find out the tumor at the preoperative marking site, we resected the transverse colon at the distance of 5 cm from the marking and dissected the marginal and intermediate lymph nodes. Consequently, partial surgical resection of the transverse colon and D2 lymph node dissection were performed.

**Fig. 6:**
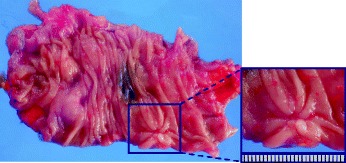
Findings of the excised specimen. The tumor was absent from the excised specimen; only an ulcer scar remained

**Fig. 7:**
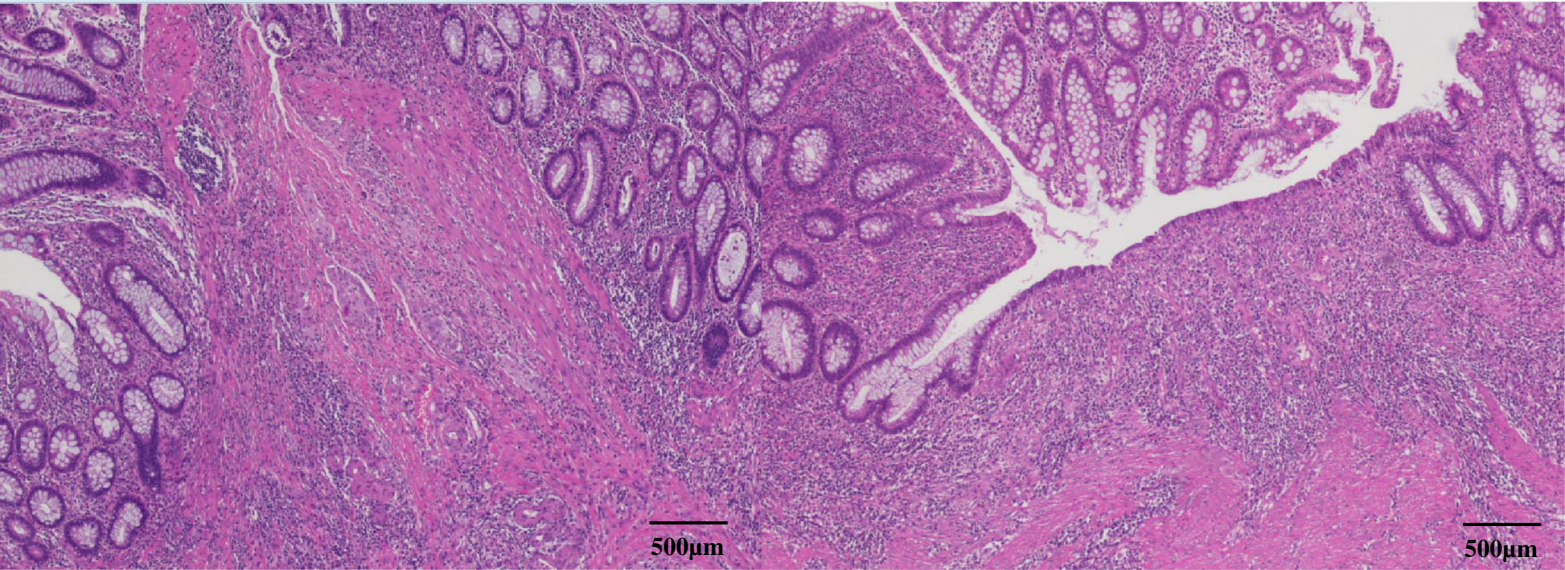
Histopathological examination of the excised specimen. Histology revealed inflammatory cell infiltration and fibrosis from the submucosal to muscularis propria layer as well as a surface covered by regenerative mucosa without a glandular cavity. No cancer cells were detected

**Fig. 8:**
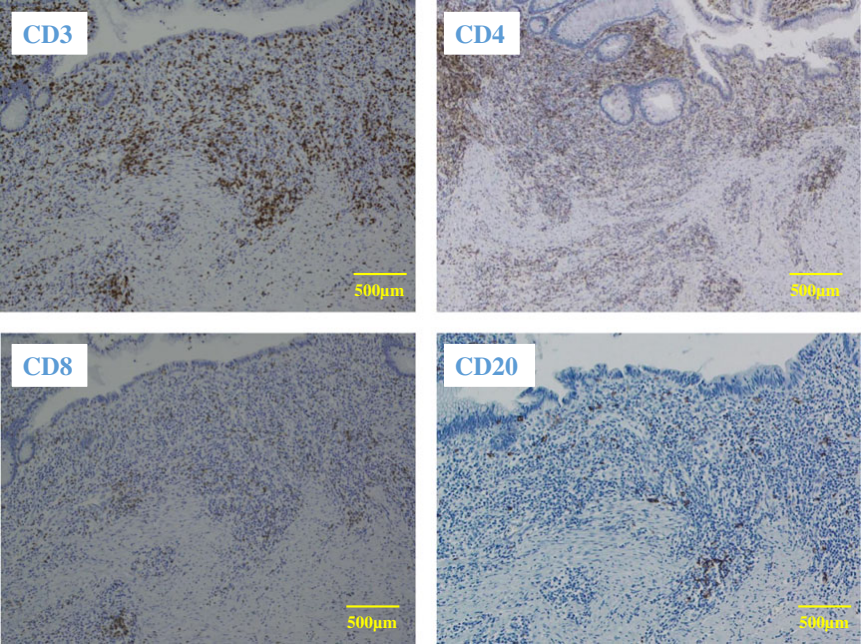
Immunohistochemical staining of the excised specimen. Immunostaining revealed a large number of CD3^+^CD4^+^ T cells, along with CD20^+^ B cells. Only a few CD8+ T cells were observed

Notably, the tumor was absent from the excised specimen, and only an ulcer scar remained (Fig 6). Histopathology revealed inflammatory cell infiltration and fibrosis from the submucosal to MP layers and a surface covered by regenerative mucosa without a glandular cavity. No cancer cells were detected (Fig. 7). Immunohistochemical staining of the excised specimen revealed a significant amount of CD3^+^CD4^+^ T cells as well as CD20^+^ B cells and a few CD8^+^ T cells (Fig. 8).

**Fig. 9:**
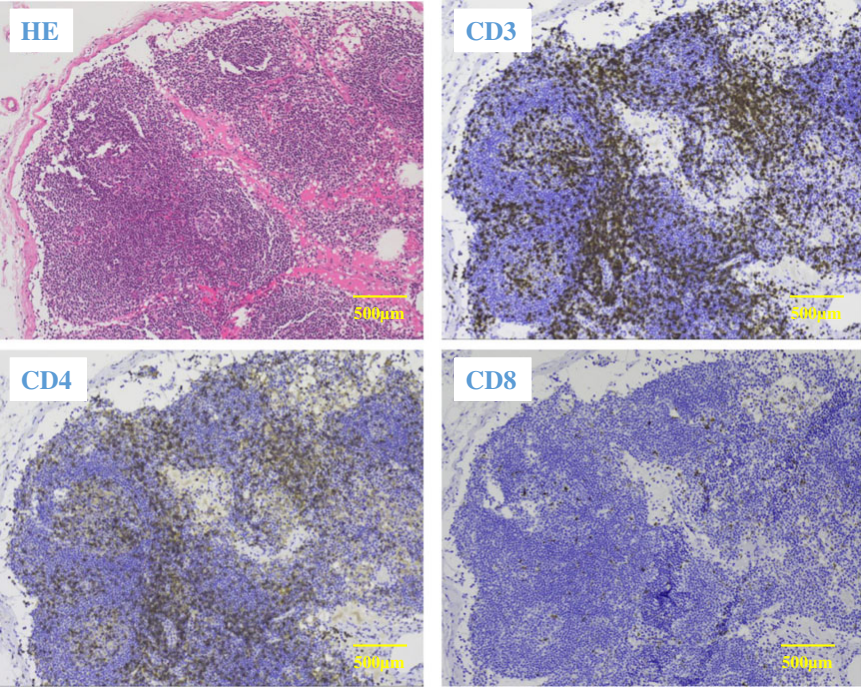
Immunohistochemical staining of the dissected lymph nodes. Immunohistochemical staining of the dissected lymph nodes revealed a significant amount of CD3 + CD4+ T cells and a few CD8+ T cells

The number of total dissected lymph nodes was eight. No lymph node metastasis was detected. Immunohistochemical staining of the dissected lymph nodes revealed a significant amount of CD3 + CD4+ T cells and a few CD8+ T cells (Fig. 9).

The final pathological staging was pStage 0 (T0N0) according to the TNM classification.

The patient had an uneventful recovery. CS performed 5 months postoperatively revealed an absence of tumors in the colon and rectum, and his CA19-9 level decreased to within the normal range. The patient remains under outpatient follow-up at 12 months postoperatively, with no observed recurrence.

### Discussion

SR of cancer is one of the most remarkable phenomena in medicine. Thus far, SR is reported in virtually all types of human cancer, with the highest numbers of reported cases among patients with neuroblastoma, renal cell carcinoma, malignant melanoma, and lymphoma/leukemia. In contrast, SR of CRC is extremely rare [4]. A review of cases involving SR of CRC between 1900 and 2005 confirmed only 11 such cases, at least with regard to the primary tumor [5].

During the current investigation, only five cases of SR of primary CRC, including our case, have been reported since 2006 (Table 1) [6–9]. In four of these five cases, SR without lymph node metastasis was pathologically proven in surgical specimens, and the duration of confirmed SR was less than 6 months. In all cases except ours, tumor reduction was revealed after biopsy, and heavy inflammatory cell infiltration was observed in the biopsy and/or excised specimens from all cases. In our case, a significant lymphocytic aggregation comprising CD3^+^CD4^+^ T cells and CD20^+^ B cells was observed around the tumor in the biopsy specimen. We, therefore, presume that a strong immune response occurred in our case as well as the other reported cases of SR of CRC.

**Table 1:** Five cases of SR of primary CRC reported since 2006

Author(year)	Age/sex	Primary site	Morphological type	Diameter (mm)	Histology	Duration (months)	Operation
			(Borrmann)				
Sakamoto(2009) [6]	80/M	Rectum	I	25	tub1	3	+
Shimizu(2010) [7]	80/M	Transverse	I	25	tub2	6	−
Sekiguchi(2013) [8]	69/F	Ascending	II	20	tub2	1.5	+
Kihara(2015) [9]	64/M	Transverse	II	30	tub2	1.5	+
Present case	80/M	Transverse	II	30	por	1	+

Regarding the surgical procedure, SR of cancer can be proven only in the excised specimens; thus, we suggest that it is necessary to resect the primary cancer with secured enough margins, and the dissection of lymph nodes should correspond to preoperative staging, even if there is SR of colon cancer.

Although many of the mechanistic details of SR of cancer remain unclear, the following have been suggested as factors: immunological, endocrine, metabolic, surgical, and postoperative events; elimination of a carcinogen or antigen; inhibition of angiogenesis; tumor necrosis; changes in oncogenes, growth factors, or cytokines; genetic and epigenetic factors; induction of benign differentiation and apoptosis; and psychological factors [3, 10]. Previous reports have described a central role for immune-mediated host responses in SR of solid tumors; for example, SR has been observed in many patients with carcinomas that contained significantly higher numbers of infiltrating immune cells (e.g., activated CD3^+^ T cells, NK cells, antigen presenting cells) than the non-regressing controls [11].

Although CRCs have long been considered poorly immunogenic, some CRCs exhibit marked inflammatory cell infiltration. In a multivariate analysis of factors affecting rectal cancer, Jass et al. previously reported that conspicuous lymphocytic infiltration along the invasive margin was an independent prognostic factor for improved survival [12]. Ropponen et al. reported that according to both univariate and multivariate analyses, the presence of tumor-infiltrating lymphocytes (TILs) is a significant predictor of improved overall and recurrence-free survival among patients with CRC [13]. Furthermore, CRCs with the high microsatellite instability (MSI-H) phenotype, which accounts for approximately 15% of sporadic CRCs, tend to associate with high numbers of TIL; accordingly, this phenotype is currently considered a clinically useful predictor of a good outcome [14]. Unfortunately, our patient refused to undergo examination for MSI status; thus, MSI status of this case was unknown.

CD8^+^ T cells have traditionally been considered the key factor in effective antitumor immunity. Because most tumors do not express major histocompatibility complex (MHC) class II, the potential antitumor-protective role of CD4^+^ T cells, which bind MHC class II molecules on target cells, has been less obvious. Nevertheless, Overwijk found that each person’s naive CD4^+^ T cells spontaneously responded to neoepitopes or peptides derived from cancer proteins encoded by mutated genes. Furthermore, these neoepitope-specific CD4^+^ T cells could directly induce cancer cell death and tumor regression by altering the tumor-promoting functions of cells in the surrounding tumor microenvironment [15].

In addition, MSI-H tumor cells have been shown to generate and present novel tumor-specific frameshift-derived neopeptides (FPS) [16]; these appear to affect the immune responses mediated by neoepitope-specific CD4^+^ T cells. In particular, pronounced CD4^+^ T cell infiltration has been observed in MSI-H CRCs that lack MHC class II expression [17]. In our case, although few infiltrating CD8^+^ T cells were observed, large numbers of CD4^+^ T cells were present along the cancer stroma in both biopsy and excised specimens and dissected lymph nodes. Our case suggests an association between SR of CRC and an adaptive immunological response to the carcinoma that is mediated particularly by CD4^+^ T cells.

## Conclusions

In conclusion, we have presented our experience with a rare case of SR of a transverse colon cancer. Our findings suggest that this SR may have been attributable to an immune-mediated antitumor response, mainly mediated by CD4^+^ T cells. SR induction may involve multiple mechanisms that, in some cases, are intricately linked. Accordingly, it may be difficult to determine the predominant mechanism in a particular case. However, we believe that the elucidation of SR-related mechanisms will lead to improved immunotherapy and cancer prevention methods.

## Additional file

Additional file 1: Consent form for case reports. (PDF 457 kb)
