# Supplementary material for: Spontaneous regression of transverse colon cancer: a case report
Source: Surg Case Rep. 2017 May 10;3:65. doi: 10.1186/s40792-017-0341-z (PMC5423878; doi:10.1186/s40792-017-0341-z)
Supplement: Additional file 1: — Consent form for case reports. (PDF 457 kb) [file 40792_2017_341_MOESM1_ESM.pdf]

CONSENT FORM FOR CASE REPORTS

For a patient's consent to publication of information about them in a journal or thesis

Name of person described in article or shown in photograph:
Eiji Toda

Title of article:_ Spontaneous regression of colon cancer.

Medical practitioner or corresponding author:__Kazuaki Nakanishi

I Eiji Toda give my consent for this information about MYSELF OR MY CHILD, relating to the subject matter above ("the Information") to appear in a journal article, or to be used for the purpose of a thesis or presentation.

I understand the following:

1. The Information will be published without my name/child's name/relatives name attached and every attempt will be made to ensure anonymity. I understand, however, that complete anonymity cannot be guaranteed. It is possible that somebody somewhere - perhaps, for example, somebody who looked after me/my child/relative, if I was in hospital, or a relative - may identify me.
2. The Information may be published in a journal which is read worldwide or an online journal. Journals are aimed mainly at health care professionals but may be seen by many non-doctors, including journalists.
3. The Information may be placed on a website.
4. I can withdraw my consent at any time before online publication, but once the Information has been committed to publication it will not be possible to withdraw the consent.

Signed: Eiji Toda Date: 2016/12/15
